# Treatment of Fangs

**Published:** 1863-05

**Authors:** Geo. F. Foote


### Correspondence.

### TREATMENT OF FANGS.

Doctor Taft: You ask me to state for the benefit of your readers how I treat and manage fangs, the nerves of which are exposed or destroyed. This I do with pleasure, and as I am an enthusiast, having my hobbies, you will indulge me in saying, "Eureka."

The course is simple and effective, even to the eradication of old alveolar abscesses in patients with good recuperative powers, where absorption of the fang has not commenced. Let us take a simple case: An exposed nerve that will not tolerate a filling over it. This we will destroy with arsenical paste, (where we can not use the broach). This paste is made by taking equal parts by weight of arsenious acid and sacharine lactis, common arsenic and sugar of milk. These are ground together in a mineral mortar for one hour; then add sufficient creosote to make a paste, and grind another hour. The arsenic by this process is minutely divided, dissolved and readily absorbed, acting promptly. Of this a minute quantity applied directly upon the nerve with a pledgit of cotton over it, destroys the sensibility (or nearly so) in a few hours, when the broach, with a transverse point, will sever its connection. Now, with a fine syringe wash out with simple tepid water all the remains of the arsenious paste and make no further applications, leaving the parts open without cotton, even for a week or ten days, when the dead nerve and lining membrane can readily be removed. After which, with the syringe, wash out repeatedly, using tepid water alone. Should the cavity be irregular, or too small to fill easily, enlarge it with a reamer, made in the form of a square broach. These are of different sizes and made of piano wire, which is sufficiently elastic and composed of the best of steel. They are readily filed down to the desired shape. Most of my fang instruments are made of this material, and made with the file and emery wheel, preserving the elastic temper, which is peculiarly adapted to this work. Another form of fang drill is made by grinding off flatly the end of a very small round wire at an angle of about 30°. Now, without further treatment, or the application of any medicine, creosote, or other escharotics, dry out the cavity and fill. The first piece of gold must go to the apex, and be there thoroughly consolidated. This is best done by a few taps of the mallet. For easy introduction, it is better to roll small ribbons of gold on broaches, carrying them with the gold to the end of the cavity, when, by a reverse motion to that used in rolling on the ribbon, it is loosened from the gold, and the broach may be used as a plugger. Fill up the fang with gold, crowding in all that it will receive of soft foil. Then fill the crown cavity immediately, *with gold only*, in the usual way, and fear no evil *ceterus paribus.*

I have yet to see the first failure in such a case.

The next in importance is when the nerve is already dead: Carefully remove all decayed matter and wash or syringe with tepid water several syringes' full at each sitting. Repeat this daily, abstaining from all other medicinal applications, and leave the cavity entirely open for the escape of matter or gasses. Each injection of water will irritate some, for it must be applied with force. If found to irritate so as make the tooth quite sore, use the water only every other day, after the first two or three. At the end of a week or ten days, or two weeks, according to the recuperative powers of the patient, it will be safe to fill the fang same as in the former case, reserving the crown filling for some subsequent day in order to avoid exciting *periostetis* by too much manipulation. Should no inflammation arise, this may be filled within two or three days. Should periosteal inflammation set in at any time after the fang is filled, apply around the gums tincture of arnica and iodine, order cold foot baths and have the patient rapidly inhale fresh air through the nostrils. The arnica and iodine stimulate absorption, the foot baths draw off the excitement from the head, while the rapid breathing sets the blood bounding to all parts of the system, and equalizes the circulation; all of which tends to lessen the congestion about the tooth.

In cases of alveolar abscess the treatment is the same, only be longer about it. It may be necessary in some cases to scrape the jaw bone externally, or even to remove some portions of it, when, by long standing, it has become involved. This may be readily done with a sharp flat excavator through the opening in the gum. The injection of cold water externally and through the fang will stimulate a sufficient reaction to secure a permanent cure in most cases.

Much time and patience is necessary to a success, both on the part of the patient and operator. Too much haste endangers the operation. After filling the fang, a temporary filling of some of the preparations of gutta percha can be made without irritation, and will preserve the tooth until all danger from periostitis is past.

Never separate teeth with the wedge or rubber, the fangs of which you are about to fill, or have recently filled.

There, sir, is the gist of the whole matter. I have practiced it successfully for three years, and the only failures I have to record have been in consequence of deviations from the above plan.

I have long since thrown aside the use of all escharotics, creosote and tannin included, except when I wish to destroy the nerve, as above stated.

The great want of success in fang filling and treating alveolar abscesses, exposed nerves, or sensitive dentine, is the result of too much "doctoring," too much "treatment," and when the profession has learned to place more confidence in the recuperative powers of the system, more faith in the *vis medicatrix naturæ*, and less in nostrums, we shall have achieved a success, and our progress will be onwards and upwards.

Yours, &c., GEO. F. FOOTE.

